# Resting-state EEG microstate dynamics reflect individual differences in tactile angle discriminability

**DOI:** 10.3389/fnins.2026.1877521

**Published:** 2026-07-29

**Authors:** Wu Wang, Yunran Guo, Kun Liang, Zikang Niu, Yulong Liu, Yang Liao, Liu Yang

**Affiliations:** 1 Department of Research, Air Force Medical Center, Air Force Medical University, Beijing, China; 2 School of Psychological and Cognitive Sciences, Peking University, Beijing, China; 3 Key Laboratory of Intelligent Monitoring on Navigation Safety, Hunan University of Information Technology, Changsha, China

**Keywords:** brain dynamics, individual differences, resting-state EEG microstates, somatosensory perception, tactile spatial acuity

## Abstract

Tactile spatial acuity, the ability to discriminate fine spatial details, is a fundamental sensory capacity modulated by both peripheral and central mechanisms. Here, we investigated whether resting-state EEG microstate dynamics are associated with individual differences in tactile spatial acuity. Following resting-state EEG recordings, 81 healthy right-handed participants performed a tactile angle discrimination (TAD) task. Five canonical microstate classes (A–E) were identified using k-means clustering (*k* = 5, explaining 78% of GFP variance). The primary analysis employed multiple linear regression with continuous TAD threshold as the outcome and microstate parameters (mean duration, occurrence, coverage) and transition probabilities as predictors, with sex included as a covariate. Regression analysis revealed that microstate A occurrence was the strongest predictor of TAD threshold (β = −0.40, *p* = 0.002), with higher occurrence associated with better tactile acuity; consistent patterns were observed for mean duration and coverage, although these parameters are algebraically interrelated with occurrence. The overall model for transition probabilities was significant (adjusted *R*^2^ = 0.422, *p* < 0.001), but no individual transition reached statistical significance. Exploratory group comparisons showed numerically consistent patterns, with the low-threshold/high-acuity group exhibiting higher occurrence and coverage of microstate A and elevated transitions involving A→B, A↔D, and B↔D, whereas the high-threshold/low-acuity group showed higher C↔E transitions. This study suggests that specific EEG microstate parameters – particularly microstate A occurrence—may serve as promising candidate neurophysiological indices of tactile spatial acuity, underscoring the role of large-scale brain network dynamics in shaping fundamental sensory function.

## Introduction

Tactile spatial acuity, which refers to the ability to discriminate fine spatial details on the skin surface, is a fundamental sensory capacity that underlies humans’ exploration of the physical world, execution of fine motor tasks, and engagement in social interactions (Craig and Kisner, 1998; Tannan et al., 2005; Peters et al., 2009; Zingaretti et al., 2019). Commonly assessed using two-point discrimination or grating orientation discrimination (Johnson and Hsiao, 1992; Craig and Johnson, 2000), tactile spatial acuity serves as a widely used index of somatosensory functional integrity. Several related, but conceptually distinct, measures have been employed, including two-point gap detection (Gibson and Craig, 2005) and identification of grating orientation (Peters et al., 2009). Classical work has further linked individual differences in acuity to cortical representation in primary somatosensory cortex (Schweizer et al., 2008) and experience-dependent plasticity (Recanzone et al., 1992). Building on this established literature, our laboratory developed the Tactile Angle Discrimination (TAD) task, a variant of the grating orientation discrimination paradigm, to provide a continuous, low-variance measure of spatial acuity suitable for individual-differences research (Wu et al., 2010; Wang et al., 2019, 2022). Prior work has validated its reliability and sensitivity across populations (Yang et al., 2014; Liu et al., 2021).

The acuity is constrained by multiple physical and physiological factors. Peripherally, physical characteristics such as fingertip size (where smaller fingertips are generally associated with higher mechanoreceptor density and superior spatial resolution), skin biomechanical properties (e.g., site, limb axis, and compliance), and stimulus parameters (e.g., contact area, pressure, vibration frequency) directly shape the initial input signal (Craig and Kisner, 1998; Tannan et al., 2005; Cody et al., 2008; Peters et al., 2009; Manfredi et al., 2012; Deflorio et al., 2023). At the neural encoding level, the type, density, distribution, and receptive field properties of low-threshold mechanoreceptors (e.g., slowly adapting type I Merkel cells and rapidly adapting Meissner’s corpuscles) constitute the first stage of spatial information processing, thereby determining the fidelity of the signal transmitted centrally (Johansson and Vallbo, 1979; Craig and Kisner, 1998; Cody et al., 2008; Deflorio et al., 2023).

However, tactile acuity cannot be fully explained by peripheral input, as the central nervous system plays a crucial and modulatory role in shaping perceptual outcomes (Peters et al., 2009; Peters and Goldreich, 2013; Deflorio et al., 2023). After initial processing in the primary somatosensory cortex (SI) via the ventral posterior lateral nucleus thalamus (Abraira and Ginty, 2013), tactile signals are subsequently processing within a distributed network, including the secondary somatosensory cortex (SII), posterior parietal cortices, insular cortex, and prefrontal regions, which are integrated and interpreted in higher-order functions such as attention, memory, multisensory integration, and perceptual decision-making (Catani et al., 2017; Sasaki et al., 2023). Critically, the intrinsic functional state of the brain (e.g., its spontaneous, resting-state dynamics) may establish a neural baseline or pre-activation level that fundamentally impacts the efficiency of tactile information processing. Naturally, this highlights the importance of investigating how large-scale, transiently stable patterns of brain network coordination, such as those captured by electroencephalographic (EEG) microstates (Britz et al., 2010; Khanna et al., 2015; Jaltare and Torta, 2024; Tarailis et al., 2024; Meynaghizadeh Zargar et al., 2025), which might be related to individual differences in tactile acuity. Therefore, studying EEG microstates offers a non-invasive window into the macroscopic neural mechanisms that may underlie the central regulation of tactile spatial acuity.

EEG microstates are brief (60–120 ms), quasi-stable topographic maps of scalp voltage, during which the global brain activity keeps relatively consistent before abruptly transitioning to a different configuration (Khanna et al., 2015; Tarailis et al., 2024). They are thought to reflect the spontaneous, coordinated activation of distributed neural assemblies, constituting the basic building blocks of human consciousness and information processing (Britz et al., 2010; Sasaki et al., 2024). Generally, previous EEG microstate studies typically report between four and six canonical classes (labelled A through F) to optimally describe resting-state EEG, explaining 65–84% of the variance in the data, respectively (Michel and Koenig, 2018; Tarailis et al., 2024). Currently, although still subject to debate, the cortical origins of these microstates are generally attributed to the activity of functional brain networks, such as microstates C and E, related to the default mode, self-experience subnetwork, and salience network, and microstates D related to attentional networks and executive functioning (Britz et al., 2010; Custo et al., 2017; Tarailis et al., 2024). Moreover, these microstates serve as a valuable complement to waveform analysis, eliminating the need for a priori selection of reference points, electrodes, or time points. Although direct applications of EEG microstate analysis to CT-optimized affective touch remain limited, the methodological framework is well established (Khanna et al., 2015), and recent studies have begun relating microstate dynamics to tactile perceptual performance (Sasaki et al., 2024), a paucity of research has specifically examined their association with tactile spatial acuity. Consequently, this study aims to explore the potential link and underlying mechanisms by which the temporal dynamics of EEG microstates, specifically their duration, occurrence, coverage, and transition probabilities, may be associated with individual differences in tactile spatial acuity.

To investigate the above questions, we adopted the tactile angle discrimination (TAD) task, following established protocols from prior research (Wang et al., 2019, 2020, 2022; Liu et al., 2021). The TAD task assesses the spatial acuity of touch perception engaging higher-order cognitive processes, such as working memory (WM) and attention. We aimed to explore the microstate disintegration under individual differences in tactile spatial acuity. Thus, following resting-state EEG recordings and the tactile acuity measurement, participants were divided into two groups based on a mean split of their TAD thresholds: a high threshold (HT, low acuity) group and a low threshold (LT, high acuity) group. Subsequently, we performed between-group comparisons of microstate metrics (e.g., duration, occurrence, coverage, transitions) to examine whether these neural dynamics differed as a function of tactile acuity and to identify potential neurophysiological indices of individual differences. We propose that the spatiotemporal dynamics of specific microstate classes may reflect the baseline efficiency of supramodal cortical networks, thereby influencing behavioral performance in tactile spatial discrimination.

## Materials and methods

### Participants

A total of 90 healthy, right-handed undergraduate and graduate students (age range: 23–32 years) were recruited for this study. All participants provided written informed consent in accordance with the guidelines approved by the Ethics Committee of the Air Force Medical Center, Air Force Medical University, and reported normal or corrected-to-normal vision, no metallic implants, and no history of neurological or psychiatric disorders, substance abuse, or recent alcohol intoxication, with their hands confirmed to be free of injuries or calluses. Participants first completed 5 min of eyes-open and 5 min of eyes-closed resting-state EEG recording, followed by the TAD task. EEG data from 9 participants were excluded from further analysis due to excessive artifacts, resulting in a final sample of 81 participants. Based on individual TAD thresholds, participants were divided into two groups using a mean split: an HT (low acuity) group (*n* = 40, age: 26.5 ± 2.4 years, 19 females) and an LT (high acuity) group (*n* = 41, age: 27.2 ± 2.6 years, 18 females).

### Tactile angle stimuli

**FIGURE 1:**
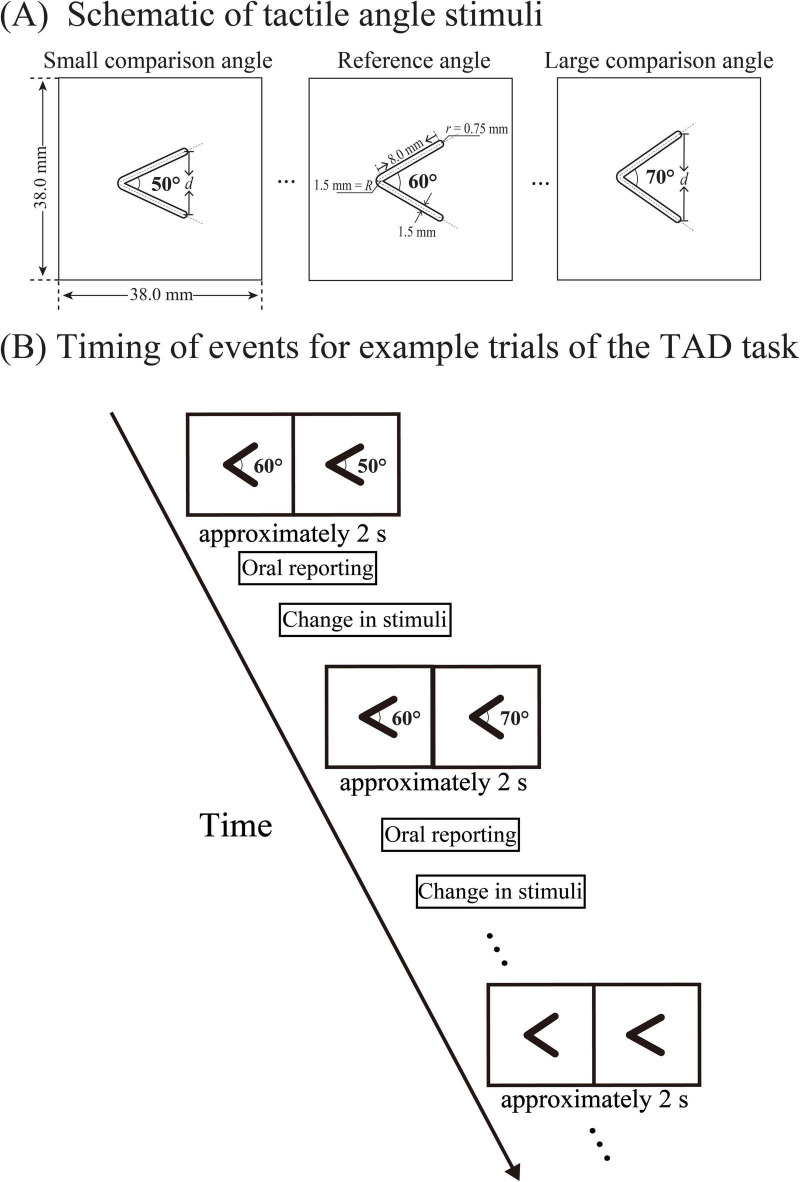
Schematic of the tactile angle stimulus and trial sequence for the TAD task. (**A**) Tactile angle stimuli. Schematic of the reference angle (60°) and two of the six comparison angles (50°, 70°). The angle is formed by two aluminum lines (8.0 × 1.5 × 0.5 mm) on a square aluminum base (38.0 × 38.0 × 2.5 mm). Key geometric parameters are indicated: endpoint distance (d), endpoint curvature radius (r), and apex curvature radius (R). (**B**) Timing of events for example, trials of the TAD task. The reference and a comparison angle were passively moved across the right index fingerpad by a manually operated slider. Participants then orally reported which angle in the pair felt larger. The experimenter recorded the response. Each of the six angle pairs was presented 20 times in a pseudorandom order, with the constraint that the same response (e.g., “reference larger” or “comparison larger”) could not occur more than twice consecutively. Additionally, the sequence of angular differences was pseudorandomized across trials, and stimulus set assignment was counterbalanced across participants.

The tactile angle stimuli were fabricated from aluminum alloy, which were similar to two-dimensional raised angles that had been employed in previous studies (Wu et al., 2010; Yang et al., 2014; Yu et al., 2018; Wang et al., 2019, 2020, 2022; Liu et al., 2021). Each stimulus consisted of two identical ridges (8.0 mm in length, 1.5 mm in width, and 0.5 mm in height) mounted on a square aluminum base (38.0 mm × 38.0 mm, 2.5 mm in height). The ridges were symmetrically positioned along an imaginary bisector, forming seven distinct included angles: 50°, 54°, 57°, 60°, 63°, 66°, and 70°. The 60° angle served as the standard reference, while the remaining angles were used as comparisons. All angles were manufactured with a tolerance of ±0.2°. Figure 1A depicts representative examples of the reference and comparison angles. The angular deviations from the reference (60°) were ±3°, ±6°, and ±10°. The corresponding distances between the outer tips of the ridges for these angle pairs were 7.6 mm and 8.4 mm (±3°), 7.3 mm and 8.7 mm (±6°), and 6.8 mm and 9.2 mm (±10°), respectively.

### TAD task

The tactile angles were delivered to the participants’ fingerpads using the Tactile Semiautomated Passive-Finger Angle Stimulator (TSPAS), as previously described (Wang et al., 2020). Each participant was blindfolded and seated at a testing table. The right hand was positioned on a hand plate, with the pad of the right index finger placed over an aperture in the plate. A pair of tactile angles, one reference and one comparison angles, was then moved horizontally (left to right) across the stationary fingerpad via a manually operated slider, traveling a distance of 76 mm at a speed of approximately 20 mm/s (Figure 1B). After perceiving the two angles in a given trial, participants were required to verbally indicate which angle felt larger. The experimenter recorded each response. This procedure was repeated across trials, with the angular pairs systematically varied. A total of 6 distinct angle pairs were presented, each repeated 20 times in a pseudorandom order. Each of the six angle pairs was presented 20 times in a pseudorandom order, with the constraint that the same response (e.g., “reference larger” or “comparison larger”) could not occur more than twice consecutively. Additionally, the sequence of angular differences was pseudorandomized across trials, and stimulus set assignment was counterbalanced across participants.

### EEG recording

EEG data were recorded using a 30-channel active electrode system (Brain Products, Germany) configured according to the international 10–20 system. Signals were acquired with the Brain Vision Recorder software at a sampling rate of 1000 Hz and were online bandpass filtered between 0.01 and 100 Hz. During recording, the online reference was set to Fz, and the ground electrode was placed at AFz (between Fz and FPz). Electrode-skin impedance was maintained below 10 kΩ for all channels throughout the experiment. The resting-state session consisted of one 5-min eyes-open and one 5-min eyes-closed block; block order was counterbalanced across participants, totaling 10 min per participant. The resting-state EEG recording (10 min total: 5 min eyes-open, 5 min eyes-closed) was followed immediately by the TAD task after a short break (<5 min). Thus, the resting-state EEG precedes the behavioral assessment within the same experimental session.

### Resting-state condition merging

Eyes-open (EO) and eyes-closed (EC) resting-state segments were concatenated prior to microstate extraction. This decision was motivated by two considerations. First, the study aimed to characterize stable, trait-like microstate organization associated with individual differences in tactile acuity, rather than state-dependent fluctuations. Pooling across EO and EC conditions yields a more representative and reliable estimate of each participant’s resting-state microstate repertoire. Second, merging the two conditions increased the total number of GFP peaks available for k-means clustering, enhancing the stability of the resulting microstate template, a practical advantage given the relatively brief (5-min per condition) recording duration. This merging approach has precedent in the literature (Custo et al., 2017; Michel and Koenig, 2018; Schumacher et al., 2019).

### EEG preprocessing

EEG data were carried out using EEGLAB(v2013.01) running under MATLAB R2013b (MathWorks, Natick, MA). Although these software versions are older, they were used because the laboratory’s established EEG preprocessing and artifact rejection pipeline, validated in prior published studies, is built on this environment, ensuring consistency and reproducibility of processing steps. The continuous recordings were first bandpass filtered (0.5–40 Hz), and a 50 Hz notch filter was applied to attenuate line noise. Subsequently, the continuous data were segmented into consecutive 2-s epochs. Artifact rejection was performed in two stages: (1) visual inspection to identify and remove epochs containing non-physiological artifacts (e.g., muscle activity, channel noise); and (2) Independent Component Analysis (ICA; Jung et al., 2001) to correct for ocular (blinks and saccades) and cardiac artifacts. Channels that were consistently noisy were corrected via 3D spherical spline interpolation. After preprocessing, a minimum of 90 artifact-free epochs per condition (eyes-closed, eyes-open) were retained for each participant. Finally, epochs from both conditions were merged into a single dataset, which was then band-pass filtered (2–20 Hz), re-referenced to the common average, and pepared with a 28-channel electrode montage (excluding FT9 and FT10) for all subsequent analyses. A representative example of the preprocessed EEG data is provided in Supplementary Figure 1 for visual inspection of data quality.

### EEG microstate analysis

**FIGURE 2:**
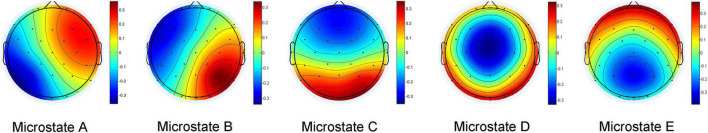
Topographies of the five identified microstate classes. The five classes that best explained the variance in the data are shown, and we labeled them microstates A to E.

Microstate analysis was conducted using Cartool (64-bit release, version 5.06.04, 2025). The software relies on the OwlNext 7.0.12 graphics library (updated 2023), ensuring modern compatibility and rendering accuracy. Consistent with established practice, topographic maps at the peaks of the global field power (GFP), the spatial standard deviation of the EEG signal across all electrodes, were extracted for clustering, as these periods represent moments of maximal topographic stability and signal-to-noise ratio (Koenig et al., 2005; Khanna et al., 2015; Michel and Koenig, 2018). A k-means clustering algorithm (Brunet et al., 2011; Pascual-Marqui et al., 1995) was applied to identify the dominant microstate topographies that best explained the global variance in the data (Pascual-Marqui et al., 1995; Brunet et al., 2011). The clustering procedure was implemented hierarchically: first, the optimal number of cluster maps was determined separately for all participants (the HT and LT groups) using a data-driven criterion; second, a common set of dominant maps was derived for all participants combined. To determine the optimal number of microstate classes (k), k-means clustering was applied for *k* = 2–8. The Krzanowski–Lai (KL) criterion and the cross-validation criterion (CVC) were evaluated; both indicated a clear elbow at *k* = 5, with diminishing returns for *k* ≥ 6. Accordingly, *k* = 5 was selected, which accounted for 78% of the GFP variance, consistent with canonical microstate literature (Michel and Koenig, 2018). Five microstate maps (labeled A–E) were identified (Figure 2), collectively explaining 78% of the global variance prior to backfitting. Subsequently, each time point of the individual preprocessed EEG was assigned to the map with which it showed the highest spatial correlation (Brunet et al., 2011). Initial cluster centers were derived by running k-means separately on randomly partitioned subsets of participants and then combined and re-optimized on the full (EO+EC merged) dataset to obtain a single, stable 5-class template. This template was then back-fitted to each participant’s EEG using the k-mean with polarity-invariant correlation. Back-fitting parameters were: Gaussian smoothing window = 10 ms, minimum microstate duration = 30 ms (≈7–8 samples at 250 Hz), and a first-order Markov assumption was used for transition probability estimation. From the backfitted microstate sequences, four conventional temporal parameters were extracted for each map: (1) global explained variance (GEV), quantifying how well a map explains the data, weighted by GFP; (2) mean duration (mDur), the average uninterrupted presence of a map in milliseconds; (3) occurrence (Occ), the frequency of appearance per second; and (4) coverage (Cov), the percentage of total analysis time occupied by a map. Additionally, transition probabilities between the five maps were examined using the Markov chain analysis (Lehmann et al., 2005).

### Electrode density consideration

Microstate analysis was performed on data recorded with a 28-channel montage (30-channel cap with FT9 and FT10 excluded). While previous studies have shown that the identification of global field power (GFP) peaks and the temporal dynamics of microstate sequences are relatively robust to moderate reductions in electrode density (Koenig et al., 2005; Khanna et al., 2015), the spatial resolution of the resulting microstate topographies is inherently limited compared to the 64–256 channel arrays typically used in canonical microstate research (Michel and Koenig, 2018; Tarailis et al., 2024). Consequently, the correspondence between the microstate classes identified here and those reported in high-density EEG studies – as well as their putative functional network associations – should be interpreted with appropriate caution.

### Data processing and analysis

To estimate the TAD thresholds, a logistic function was employed, consistent with the approach adopted in our prior research (Wang et al., 2019, 2022). The logistic function is widely utilized in psychophysical studies for threshold estimation (Hoehler, 1995; Weder et al., 1998; Kuehn et al., 2017) and is expressed as follows:


$$y=\frac{1}{1+e^{-\left(\alpha +\beta \,x\right)}}$$

where *x* is the angular difference (°), α is the intercept, and β is the slope. The tactile angle discrimination (TAD) threshold was defined as the angle difference corresponding to 50% correct performance, i.e., the point of subjective equality (PSE), estimated as *x*_50_ = −α/β. This was obtained from the fitted function by interpolating the stimulus value at which the predicted performance reached 0.5, equivalent to the midpoint between the 25% and 75% correct points. This PSE-based definition was chosen because it provides a symmetric, unbiased estimate of discriminative acuity that is less sensitive to lapse rate assumptions than a fixed 75% correct criterion.

Before performing analysis of variance (ANOVA), the normality of the data distribution was assessed using a one-sample Kolmogorov–Smirnov (K–S) test. Normality was further supported by visual inspection of Q–Q plots, in which data points were observed to align closely with the reference line. Following these checks, ANOVA was conducted using the bruceR package in R programming, with post hoc comparisons performed via Tukey’s honestly significant difference (HSD) method (Bao, 2021). The primary analytical approach treated the TAD threshold as a continuous variable to preserve individual variability. Multiple linear regression models assessed the association between TAD threshold and each microstate parameter (mean duration, occurrence, and coverage) and transition probabilities. For group comparison ANOVAs, participants were additionally divided into HT and LT groups via a mean split. Exploratory mixed-design ANOVAs were conducted with a 2 (group: HT vs. LT) × 5 (microstate class) design for microstate parameters, and a 2 (group) × 20 (transition probability pairs) design for transition probabilities. These ANOVAs are presented solely for descriptive purposes and are not used for primary hypothesis testing. To account for the multiple comparisons arising from the large number of tests across microstate parameters and transition probabilities, we applied the Benjamini–Hochberg false discovery rate (FDR) correction (Benjamini and Hochberg, 1995) separately to two conceptually distinct families of comparisons: (a) post-hoc pairwise contrasts across microstate classes (for each of the four parameters), and (b) pairwise comparisons among transition probabilities. This approach follows recommendations to apply multiplicity adjustments within well-defined families of inferences (Proschan and Waclawiw, 2000). Both raw *p*-values and FDR-adjusted *q*-values are reported in the Supplementary Materials.

## Results

### Resting-state EEG activity

The five microstate maps from all participants in the combination of the eyes-open and closed conditions are shown in Figure 2. These five maps are largely similar to the one described previously (Michel and Koenig, 2018; Zanesco et al., 2020; Tarailis et al., 2024; Wang et al., 2024) and labelled as maps A, B, C, D, and E. Note that microstate parameters are algebraically interrelated: coverage ≈ occurrence × mean duration, and GEV depends on the GFP magnitude of the assigned segments. Therefore, significance across multiple parameters does not constitute independent statistical evidence. In keeping with common practice (Michel and Koenig, 2018), occurrence was treated as the primary inferential parameter, with mean duration and coverage reported for descriptive purposes.

### Primary analysis: continuous regression

The primary analytical approach treated the TAD threshold as a continuous variable to preserve individual variability. Multiple linear regression models assessed the association between TAD threshold and each microstate parameter (mean duration, occurrence, coverage) and transition probabilities, with sex included as a covariate.

### Mean duration

A multiple linear regression was conducted with TAD threshold as the dependent variable and the mean duration of each microstate class (A–E) and sex as predictors. The overall model was significant (*F*_6, 74_ = 4.27, *p* < 0.001, adjusted *R*^2^ = 0.20), indicating that microstate mean duration jointly accounted for approximately 20% of the variance in tactile acuity. Among the individual predictors, Microstate A emerged as the most robust predictor (β = −0.55, *p* < 0.001, 95% CI [−0.26, −0.07]), with longer mean duration of Microstate A associated with lower TAD threshold (i.e., better tactile acuity). Microstate B (β = 0.36, *p* = 0.038, 95% CI [0.01, 0.31]) and Microstate C (β = 0.21, *p* = 0.043, 95% CI [0.002, 0.14]) also showed significant positive associations with TAD threshold, indicating that longer mean duration of these classes was related to poorer tactile acuity. Microstate D (β = −0.22, *p* = 0.151, 95% CI [−0.15, 0.02]) and Microstate E (β = 0.06, *p* = 0.69, 95% CI [−0.07, 0.10]) did not reach significance. Sex was not a significant predictor (β = 0.05, *p* = 0.82, 95% CI [−0.65, 0.82]). Regression diagnostics indicated acceptable multicollinearity (all VIFs < 3), homoscedasticity (Breusch–Pagan test, p = 0.36), and no extreme influential cases exceeding the 4/n threshold after excluding six borderline observations (Cook’s *D* < 0.05). However, the Shapiro–Wilk test indicated a slight deviation from normality (*W* = 0.95, *p* = 0.005); given the moderate sample size, this is unlikely to bias the estimates substantially. Full regression results are summarized in Table 1.

**TABLE 1:** Multiple linear regression predicting TAD threshold from mean duration of microstate classes and sex.

Predictor	β	*B* (ms)	*SE*	*t*	*p*	95% CI for *B*
(Intercept)	–	4.25	2.79	1.52	0.132	[−1.31, 9.80]
Microstate A	**-0.55**	−0.17	0.05	−3.50	**<0.001**	[−0.26, −0.07]
Microstate B	**0.36**	0.16	0.07	2.12	**0.038**	[0.01, 0.31]
Microstate C	**0.21**	0.07	0.03	2.05	**0.043**	[0.002, 0.14]
Microstate D	−0.22	−0.06	0.04	−1.45	0.151	[−0.15, 0.02]
Microstate E	0.06	0.02	0.04	0.41	0.685	[−0.07, 0.10]
Sex	0.05	0.08	0.37	0.22	0.824	[−0.65, 0.82]

β = standardized coefficient; B = unstandardized coefficient (ms); SE = standard error; CI = confidence interval. Significant predictors (*p* < 0.05) are bolded.

### Occurrence

The second multiple linear regression was conducted with TAD threshold as the dependent variable and the occurrence of each microstate class (A–E) and sex as predictors. The overall model was significant (*F*_6, 74_ = 6.28, *p* < 0.001, adjusted *R*^2^ = 0.284), indicating that microstate occurrence jointly accounted for approximately 28% of the variance in tactile acuity. Among the individual predictors, Microstate A emerged as the strongest predictor (β = −0.40, *p* = 0.002, 95% CI [−2.08, −0.50]), with higher occurrence of Microstate A associated with lower TAD threshold (i.e., better tactile acuity). Microstate C also showed a significant positive association with TAD threshold (β = 0.25, *p* = 0.022, 95% CI [0.09, 1.16]), indicating that higher occurrence of this class was related to poorer tactile acuity. Microstate B (β = 0.10, *p* = 0.410, 95% CI [−0.52, 1.26]), Microstate D (β = −0.20, *p* = 0.055, 95% CI [−1.36, 0.02]), and Microstate E (β = 0.14, *p* = 0.211, 95% CI [−0.25, 1.12]) did not reach significance. Sex was not a significant predictor (β = 0.07, *p* = 0.718, 95% CI [−0.57, 0.82]). Regression diagnostics indicated acceptable multicollinearity (all VIFs < 1.71), homoscedasticity (Breusch–Pagan test, *p* = 0.744), and no extreme influential cases exceeding the 4/n threshold (Cook’s *D* < 0.05), with only four borderline observations identified. The Shapiro–Wilk test indicated a slight deviation from normality (*W* = 0.97, *p* = 0.038); given the moderate sample size, this is unlikely to bias the estimates substantially. Full regression results are summarized in Table 2.

**TABLE 2:** Multiple linear regression predicting TAD threshold from the occurrence of microstate classes and sex.

Predictor	β	*B* (ms)	*SE*	*t*	*p*	95% CI for *B*
(Intercept)	–	6.39	1.88	3.40	0.001	[2.64, 10.13]
Microstate A	**-0.40**	−1.29	0.40	−3.25	**0.002**	[−2.08, −0.50]
Microstate B	0.10	0.37	0.45	0.83	0.410	[−0.52, 1.26]
Microstate C	**0.25**	0.63	0.27	2.34	**0.022**	[0.09, 1.16]
Microstate D	−0.20	−0.67	0.35	−1.95	0.055	[−1.36, 0.02]
Microstate E	0.14	0.44	0.35	1.26	0.211	[−0.25, 1.12]
Sex	0.07	0.13	0.35	0.36	0.718	[−0.57, 0.82]

β = standardized coefficient; B = unstandardized coefficient (occurrence per second); SE = standard error; CI = confidence interval. Significant predictors (*p* < 0.05) are bolded.

### Coverage

The third multiple linear regression was conducted with TAD threshold as the dependent variable and the time coverage of each microstate class (A–E) and sex as predictors. The overall model was significant (*F*_6, 74_ = 3.68, *p* = 0.003, adjusted *R*^2^ = 0.171), indicating that microstate coverage jointly accounted for approximately 17% of the variance in tactile acuity. Among the individual predictors, Microstate A emerged as the strongest predictor (β = −0.46, *p* = 0.003, 95% CI [−2.66, −0.57]), with higher coverage of Microstate A associated with lower TAD threshold (i.e., better tactile acuity). Microstate C also showed a significant positive association with TAD threshold (β = 0.29, *p* = 0.020, 95% CI [0.11, 1.29]), indicating that higher coverage of this class was related to poorer tactile acuity. Microstate B (β = 0.09, *p* = 0.501, 95% CI [−0.55, 1.11]), Microstate D (β = −0.16, *p* = 0.155, 95% CI [−1.12, 0.18]), and Microstate E (β = 0.11, *p* = .343, 95% CI [−0.33, 0.93]) did not reach significance. Sex was not a significant predictor (β = 0.06, *p* = 0.801, 95% CI [−0.61, 0.79]). Regression diagnostics indicated acceptable multicollinearity (all VIFs < 1.73), homoscedasticity (Breusch–Pagan test, *p* = 0.61), and no extreme influential cases exceeding the 4/*n* threshold (Cook’s *D* < 0.05), with only three borderline observations identified. The Shapiro–Wilk test indicated no significant deviation from normality (*W* = 0.99, *p* = 0.22). Full regression results are summarized in Table 3.

**TABLE 3:** Multiple linear regression predicting TAD threshold from time coverage of microstate classes and sex.

Predictor	β	*B* (ms)	*SE*	*t*	*p*	95% CI for *B*
(Intercept)	–	5.92	1.77	3.35	0.001	[2.39, 9.44]
Microstate A	**-0.46**	−1.62	0.53	−3.08	**0.003**	[−2.66, −0.57]
Microstate B	0.09	0.28	0.42	0.68	0.501	[−0.55, 1.11]
Microstate C	**0.29**	0.70	0.30	2.37	**0.020**	[0.11, 1.29]
Microstate D	−0.16	−0.49	0.43	−1.54	0.128	[−1.85, 0.84]
Microstate E	0.11	0.30	0.32	0.91	0.365	[−0.69, 0.97]
Sex	0.00	0.006	0.024	0.25	0.801	[−0.042, 0.054]

β = standardized coefficient; B = unstandardized coefficient (%); SE = standard error; CI = confidence interval. Significant predictors (*p* < 0.05) are bolded.

### Transition probabilities

The final multiple linear regression was conducted with TAD threshold as the dependent variable and the 20 directed transition probabilities (pair: A→B, A→C, A→D, A→E, B→A, B→C, B→D, E→A, etc.) and sex as predictors. The overall model was significant (*F*_6, 74_ = 3.70, *p* < 0.001, adjusted *R*^2^ = 0.42), indicating that the set of transition probabilities jointly accounted for approximately 42% of the variance in tactile acuity. However, no individual transition probability reached statistical significance (all *p* > 0.05), likely due to the large number of predictors relative to the sample size and the intercorrelations among transition probabilities. Sex was also not a significant predictor (β = −0.24, *p* = 0.52, 95% CI [−0.99, 0.51]). Regression diagnostics indicated acceptable normality of residuals (Shapiro–Wilk test: *W* = 0.98, *p* = 0.088) and homoscedasticity (Breusch–Pagan test: BP = 24.67, *p* = 0.22). Four observations exceeded the Cook’s distance threshold of 4/n (0.049), with maximum Cook’s D = 0.169; removing these cases did not materially alter the pattern of results. Given the large number of predictors (21) relative to the sample size (81), the adjusted *R*^2^ of 0.42 should be interpreted cautiously, and the null findings for individual predictors may reflect limited power rather than the absence of true associations.

### Exploratory group comparison (ANOVA)

For the group comparisons, mixed-design ANOVAs were conducted with a 2 (group: HT vs. LT) × 5 (microstate class) design for microstate parameters and a 2 (group: HT vs. LT) × 20 (transition probability pairs) design for transition probabilities. Detailed results are provided in the Supplementary Material. These ANOVAs are presented solely for descriptive purposes and are not used for primary hypothesis testing.

## Discussion

Our study aimed to investigate whether resting-state EEG microstate dynamics are associated with individual differences in tactile spatial acuity, as measured by the tactile angle discrimination (TAD) task. We clustered resting-state EEG data from 81 healthy adults into five microstate classes (A–E; Figure 2). The five microstate classes identified in the present study were provisionally matched to canonical microstate templates reported in high-density EEG literature (Michel and Koenig, 2018). However, given the limited spatial resolution of the 28-channel montage, these correspondences should be regarded as approximate rather than definitive, and the functional network attributions discussed below are tentative.

### Microstate A and tactile acuity

Our principal finding is that continuous TAD threshold was significantly predicted microstate A occurrence (β = −0.40, *p* = 0.002, adjusted *R*^2^ = 0.284), with higher occurrence of microstate A associated with better tactile acuity (i.e., lower TAD threshold). Consistent patterns were observed for mean duration and coverage, although these parameters are algebraically interrelated with occurrence (coverage ≈ occurrence × mean duration) and are reported for descriptive completeness (see Results). The corresponding exploratory group comparison (HT vs. LT; Supplementary Material) showed a numerically consistent pattern, further supporting the regression-based finding.

One possible interpretation is that microstate A is associated with a baseline state of heightened readiness in neural circuits relevant to fine-grained spatial analysis, which may covary with the efficiency of tactile feature encoding. Although microstate A is frequently linked to bilateral fronto-temporal networks implicated in phonological or visuospatial processing (Britz et al., 2010; Milz et al., 2016; Custo et al., 2017; Tarailis et al., 2024), this correspondence is provisional given the limited spatial resolution of the 28-channel montage; the following functional interpretations are framed at the level of broad network systems rather than specific anatomical structures. These attributions are based on topographic similarity to canonical microstate templates reported in high-density EEG literature and should be regarded as provisional. Rather than performing primary somatosensory processing, microstate A may exert its influence indirectly through interactions with multisensory association areas, such as the posterior parietal cortex (Avillac et al., 2004; Pasalar et al., 2010), where it is associated with the integration or gating of information relevant to somatosensory spatial discrimination. Therefore, a greater temporal presence of this network covaries with superior tactile perceptual performance.

Although the present findings pertain to large-scale cortical network dynamics captured by EEG microstates, converging evidence indicates that state-dependent modulation also operates at finer spatial scales. In intracellular recordings from rat somatosensory cortex, Norrlid et al. (2021) showed that identical tactile input patterns evoked multiple discrete response types depending on the time-varying state of local subnetworks, rather than being determined solely by global cortical Up/Down states. Such local state transitions are thought to be nested within, and partially shape, the expression of macroscale network modes indexed by EEG microstates. A key distinction, however, is that microstates reflect momentary global configurations of distributed neuronal assemblies rather than focal laminar or columnar activity; thus, the present results are best interpreted as reflecting the system-level instantiation of state-dependent sensory readiness, which may be underpinned by – but is not reducible to – local microcircuit dynamics (Norrlid et al., 2021).

### Transition dynamics

Regarding transition dynamics, the overall regression model including all 20 directed transition probabilities significantly predicted TAD threshold (adjusted *R*^2^ = 0.422, *p* < 0.001), indicating that the collective pattern of microstate transitions is meaningfully associated with individual differences in tactile acuity. However, no individual transition probability reached statistical significance, likely due to the large number of predictors (21) relative to the sample size (*N* = 81) and intercorrelations among transitions. This pattern suggests that the transition–acuity relationship is distributed across multiple pathways rather than driven by isolated pairs.

The exploratory group comparisons (Supplementary Material) revealed that the LT group showed numerically higher transition probabilities for A↔D, A→B, and B↔D, whereas the HT group showed higher C↔E transitions. These descriptive patterns are broadly consistent with the regression trend and may reflect differences in the balance between externally oriented (attention/sensorimotor) and internally oriented (default mode) network dynamics. Given that microstate D is frequently linked to the dorsal attention network (Custo et al., 2017; Seitzman et al., 2017), its numerically stronger coupling with microstates A and B in the LT group may reflect a brain state more conducive to sensorimotor integration and spatial attention. Conversely, the HT group’s numerically higher C↔E transitions, a pattern often associated with networks subserving self-referential or affective processing (Schiller et al., 2024; Tarailis et al., 2024), may indicate a baseline state more dominated by internal mentation, potentially at the expense of efficient processing of external sensory stimuli. Nevertheless, given the lack of individually significant predictors in the primary regression analysis, these group-level observations should be interpreted with caution and require replication in larger samples.

It is important to note that the TAD task is not a pure measure of peripheral tactile acuity but a passive, delayed-comparison paradigm requiring tactile working memory, sustained attention, and perceptual decision-making (Figure 1B). This cognitive context provides a natural explanation for the observed involvement of microstate D, which is canonically associated with the dorsal attention network (DAN) and executive control processes (Custo et al., 2017; Seitzman et al., 2017). The numerically stronger A↔D and B↔D transitions in the LT (high-acuity) group may therefore reflect a resting-state network configuration that is more conducive to engaging the attentional and mnemonic resources necessary for the task, rather than purely sensory facilitation. In this view, resting-state microstate dynamics, particularly the flexibility of transitions between sensory-attentional (A, B) and executive-control (D) networks, may prime the cognitive infrastructure of the task, complementing the sensory-level advantages indexed by microstate A occurrence alone.

### Lateralization

Microstate A exhibited a modest left-anterior/lateralized topography. Given that tactile stimulation was applied to the right index finger in right-handed participants, implying predominant left-hemisphere somatosensory processing, his asymmetry is broadly compatible with the expected lateralization of tactile sensory encoding. However, EEG microstate topographies reflect global functional network configurations rather than focal sensory responses; thus, a strict one-to-one correspondence should not be overinterpreted, particularly given the limited spatial resolution of the 28-channel montage.

### Relation to prior work

Our findings extend the growing literature on resting-state EEG microstates as markers of individual perceptual differences. While previous studies have largely focused on cognitive or clinical domains (Zappasodi et al., 2019; Jabès et al., 2021; Wang et al., 2024; Leupin and Britz, 2025; Meynaghizadeh Zargar et al., 2025), we demonstrate that microstate parameters are associated with basic tactile function. Specifically, the relationship between microstate A occurrence and TAD thresholds supports theoretical models in which resting-state brain dynamics, or “neurophysiological baselines,” substantially covary with perceptual outcomes (Adhikari et al., 2014; Saito et al., 2025). The consistency of this relationship across continuous regression and exploratory group comparisons further suggests a continuum of neurodynamic efficiency, rather than a categorical distinction between groups. Together, these results offer a novel, systems-level account of individual differences in tactile perception, complementing established peripheral and structural factors such as receptor density and thalamocortical connectivity.

### Limitations

Several limitations merit consideration. First, the sample was comprised exclusively of young, healthy adults, which restricts the generalizability of the findings to other populations, such as older individuals or clinical groups. Second, as the study is cross-sectional, all inferences regarding microstate–behavior relationships are confined to associations; causal interpretations regarding directionality or mechanism await future longitudinal or interventional designs. Third, a further limitation is the use of a 28-channel EEG montage, which provides lower spatial resolution than the 64–256 channel arrays common in microstate methodology. Although GFP-based segmentation is reasonably preserved, fine-grained topographic validation and confident attribution to specific functional networks (e.g., DMN, salience, DAN) require high-density recordings. The functional interpretations offered here are therefore tentative and await confirmation in higher-density replications. Fourth, fingertip size – a known anatomical predictor of tactile spatial acuity (Peters et al., 2009) – was not measured in the present study. Future studies should include this anthropometric covariate (e.g., distal phalanx width measured with digital calipers) to more fully disentangle peripheral anatomical contributions from central microstate-related effects. Future research should integrate EEG microstate analysis with task-based fMRI to delineate the underlying neural networks with greater spatial precision, employ longitudinal or interventional designs to examine how microstate dynamics evolve with tactile perceptual training, and explore these properties in populations with sensory impairments or across different stages of the lifespan.

## Conclusion

In summary, this study demonstrates that individual differences in tactile spatial acuity are associated with distinct spatiotemporal dynamics of resting-state EEG microstates. Specifically, microstate A occurrence was the strongest predictor of TAD threshold, with higher occurrence associated with better tactile acuity. Exploratory group comparisons further suggested a pattern of altered transition dynamics, with the LT group showing numerically higher A↔D, A→B, and B↔D transitions, and the HT group showing higher C↔E transitions. These findings suggest that EEG microstate parameters, particularly microstate A occurrence, may serve as promising, non-invasive candidate indices for evaluating tactile sensory function and elucidating the neural mechanisms underlying perceptual abilities.

## Data Availability

The datasets used to support the findings of this study are available from the corresponding author on reasonable request.
