# Supplementary material for: Resting-state EEG microstate dynamics reflect individual differences in tactile angle discriminability
Source: Front Neurosci. 2026 Jul 29;20:1877521. doi: 10.3389/fnins.2026.1877521 (PMC13483064; doi:10.3389/fnins.2026.1877521)
Supplement: Supplementary file 1 [file Presentation_1.pdf]

1. Microstate analysis pipeline description.

The microstate analysis followed an eight-step pipeline:

1. **Preprocessing**: EEG data were band-pass filtered (2–20 Hz) and downsampled to 250 Hz.
2. **GFP computation**: Global Field Power was computed for each time point.
3. **Selection of GFP peaks**: Microstate segmentation was performed exclusively on GFP peak samples.
4. **k-means clustering**: Clustering was applied for k = 2–8. The Krzanowski–Lai (KL) criterion and cross-validation criterion (CVC) both indicated an elbow at k = 5, which accounted for 78% of the GFP variance.
5. **Template initialization**: Initial cluster centers were derived by running k-means on randomly partitioned participant subsets.
6. **Final template fitting**: Subset-derived templates were combined and re-optimized on the full dataset (eyes-open and eyes-closed conditions merged) to obtain a single stable 5-class template.
7. **Back-fitting**: The template was back-fitted to each participant's EEG using the Atomize and k-means algorithm with polarity-invariant correlation. Parameters: Gaussian smoothing window = 10 ms, minimum microstate duration = 30 ms (~7–8 samples at 250 Hz).
8. **Parameter computation**: Microstate parameters (global explained variance, mean duration, occurrence, coverage) and transition probability matrices (first-order Markov assumption) were computed from the labeled time series.

2. A representative resting-state EEG data from a single participant

(A)

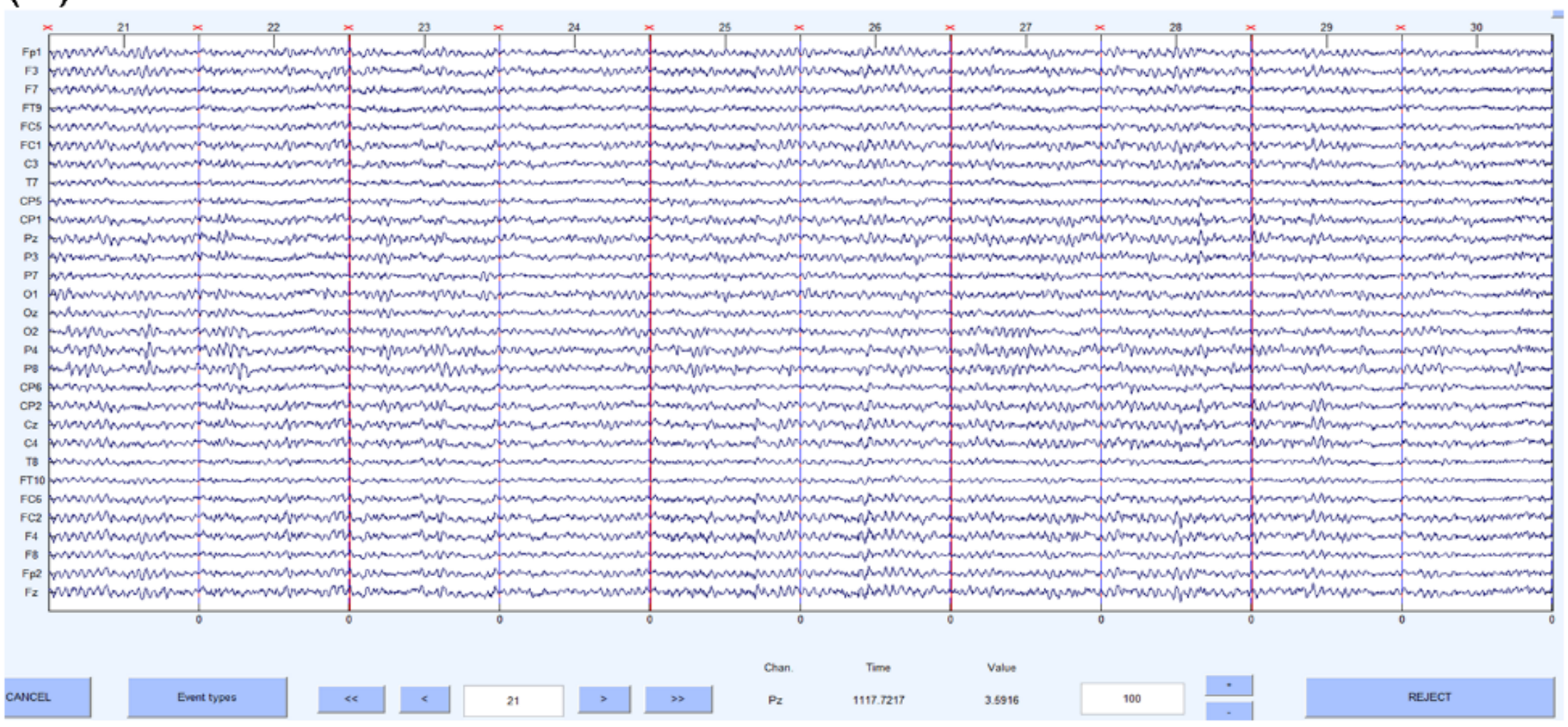

(B)

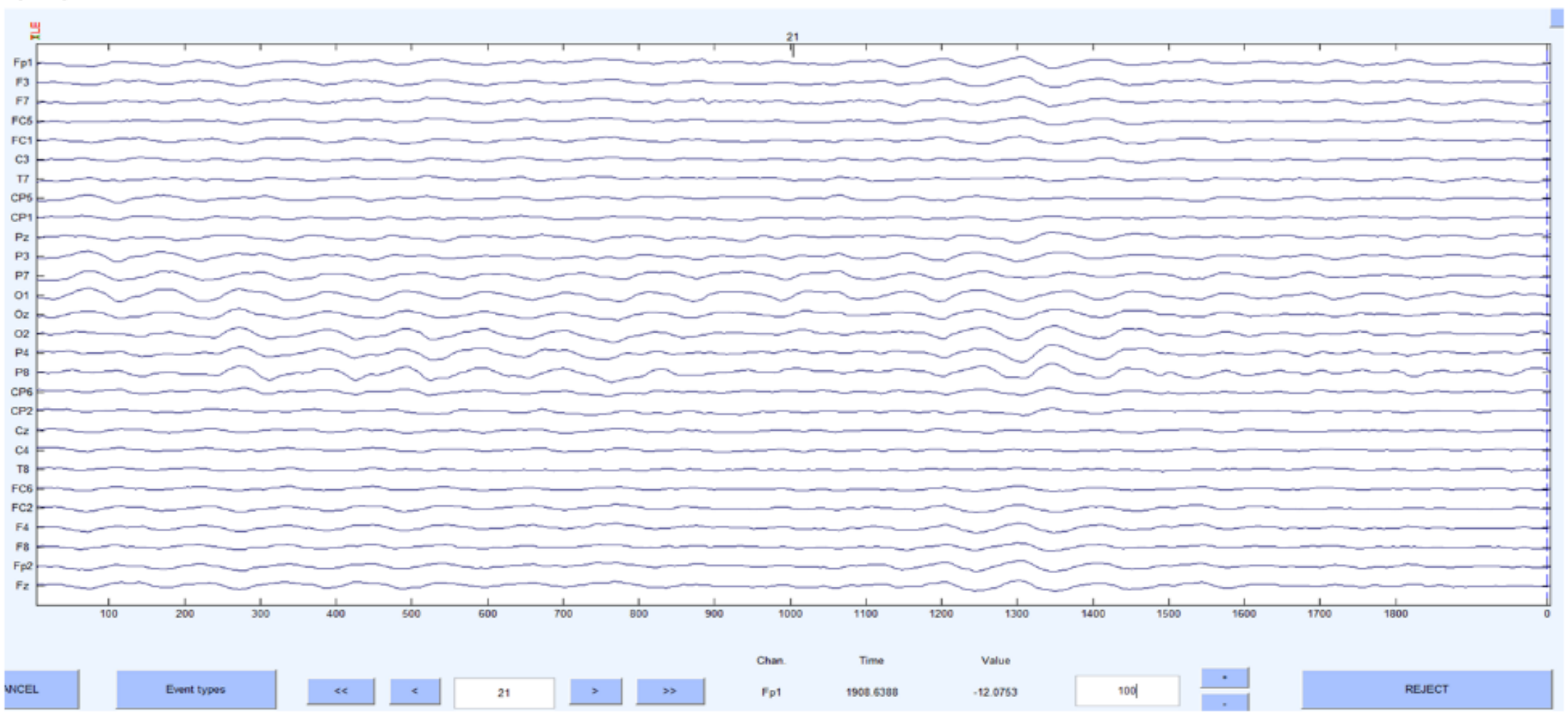

Supplementary Figure S1. Representative resting-state EEG data from a single participant (female, age 29). (A) Ten-second segment of preprocessed data (28-channel montage, 2–20 Hz band-pass filter, common average reference). (B) Enlarged 2-second segment from the boxed region in (A), illustrating typical signal quality and the absence of prominent artifacts.

3. Exploratory group comparison (ANOVA)

No formal a priori power analysis was conducted; however, a post-hoc sensitivity analysis indicated that with N = 81 (HT: n= 40; LT: n= 41), $\alpha = .05$ (two-tailed), the study had 80% power to detect Cohen's $d \geq 0.44$ in two-group comparisons and partial $\eta^2 \geq .06$ in ANOVA models. Effects smaller than this should be interpreted with appropriate caution.

Global Explained Variance: GEV. To estimate the difference in the GEV between the HT group and LT group across the five microstate classes (A to E), we performed a 2 (group: HT vs. LT) × 5 (class: A to E) mixed factor repeated-measures ANOVA with the GEV as the dependent measure and sex as a covariate. Mauchly's test of sphericity was performed, and Greenhouse–Geisser corrected F-values and degrees of freedom are reported when sphericity was violated. The results showed significant main effects of sex ($F_{1, 78} = 5.43$, $p < 0.05$, $\eta^2_p = 0.07$) and microstate class ($F_{2.08, 162.25} = 120.46$, $p < 0.001$, $\eta^2_p = 0.61$). However, the interaction between group and class was not significant ($F_{2.08, 162.25} = 1.25$, $p = 0.29$, $\eta^2 p = 0.016$). A follow-up test (Tukey's HSD) revealed a significant difference between the HT and LT groups, specifically for microstate class A ($t_{78} = 2.64$, $p = 0.011$, FDR-adjusted q = 0.11, *Cohen's d* = 0.23, Figure S2 A).

Mean Duration: mDur. We conducted a similar analysis to examine the difference in mDur between groups across microstate classes. Mauchly's test of sphericity was performed, and Greenhouse–Geisser corrected F-values and degrees of freedom are

reported when sphericity was violated. The ANOVA showed significant main effects of sex ($F_{1, 78}$ = 4.29, p < 0.05, η^2_p = 0.05) and microstate class ($F_{2.97, 231.92}$ = 88.13, p < 0.001, η^2_p = 0.53). There was no significant main effect of group, nor a significant group × class interaction ($F_{2.97, 231.92}$ = 1.36, p = 0.26, $\eta^2 p$ = 0.017), across the five classes (Figure S2 B).

Occurrence: Occ. A mixed repeated-measures ANOVA was also performed for the Occ. Mauchly's test of sphericity was performed, and Greenhouse–Geisser corrected F-values and degrees of freedom are reported when sphericity was violated. The results indicated significant main effects of sex ($F_{1, 78}$ = 4.52, p < 0.05, η^2_p = 0.06) and microstate class ($F_{3.30, 257.01}$ = 51.81, p < 0.001, η^2_p = 0.40) and, importantly, a statistically significant group × class interaction ($F_{3.30, 257.01}$ = 3.19, p < 0.05, η^2_p = 0.04). For microstate class A, the difference (Tukey's HSD) between the HT and LT groups was also significant (t_{78} = 2.76, p = 0.007, FDR-adjusted q = 0.11, *Cohen's d* = 0.43, Figure S2 C).

Coverage: Cov. The same analysis for Cov revealed a significant main effect of microstate class ($F_{3.02, 235.26}$ = 67.55, p < 0.001, η^2_p = 0.46, Greenhouse–Geisser corrected), with the sex effect ($F_{1, 78}$ = 0.66, p = 0.42) nonsignificant. The group × class interaction, however, was not significant ($F_{3.02, 235.26}$ = 2.08, p = 0.10, $\eta^2 p$ = 0.026). For microstate class A, a significant difference (Tukey's HSD) between the HT and LT groups was observed (t_{78} = 2.27, *p = 0.026, FDR-adjusted q = 0.17, Cohen's d* = 0.32,

Figure S2 D).

Tactile spatial acuity. We used the TAD threshold to assess the tactile spatial acuity. Participants were divided into two groups based on a mean (5.55 ±1.76°) split of their TAD thresholds: a high threshold (HT, low acuity) group (7.00 ±1.09°, Fig. 3 E) and a low threshold (LT, high acuity) group (4.07 ±0.83°, Figure S2 E).

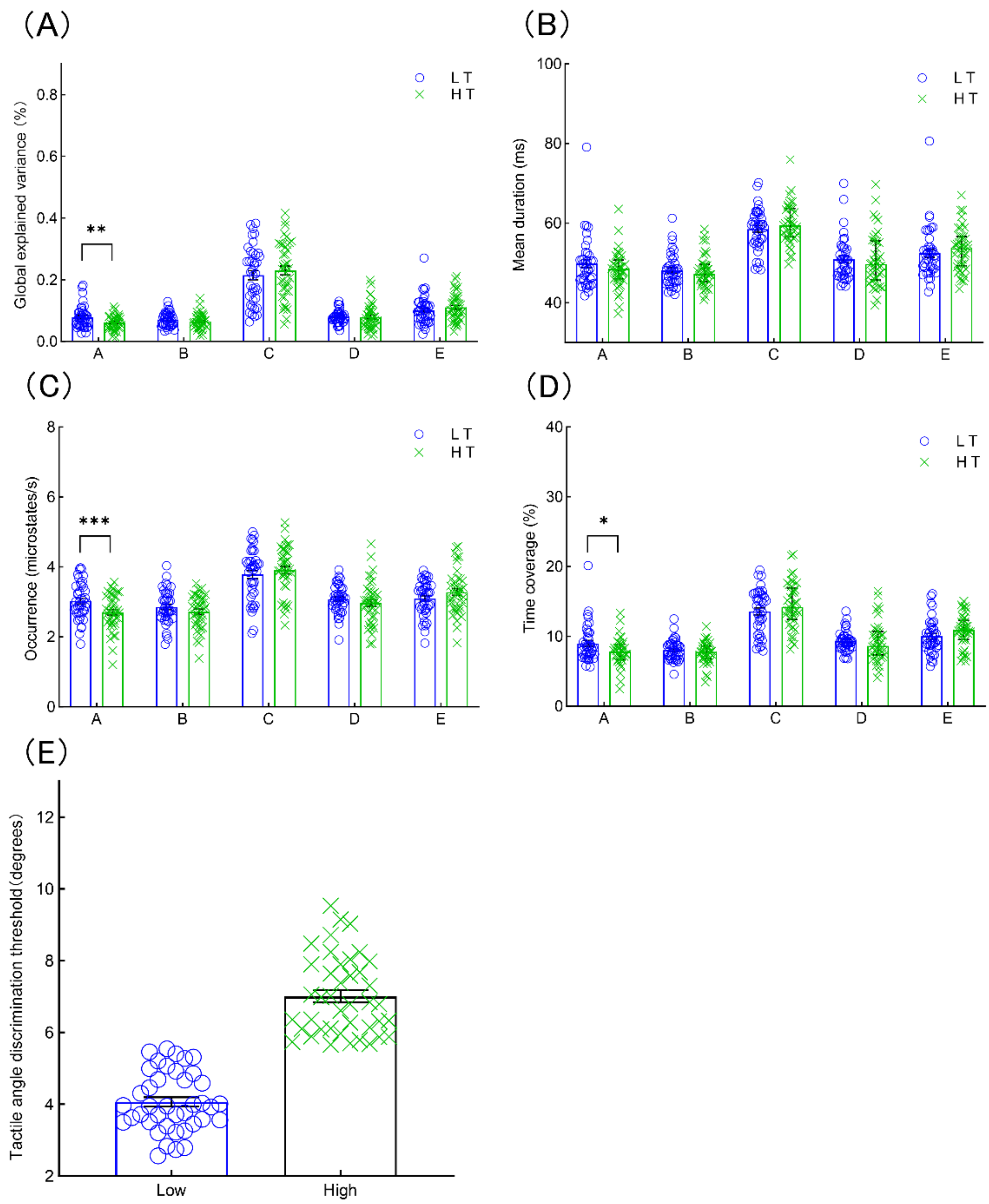

Supplementary Figure S2. Results of resting-state EEG microstates. A, GEV. The GEV of microstate A was significantly higher in the LT group than in the HT group. No significant differences in GEV were found for the remaining microstates between the two groups. B, mDur. No significant differences in mDur were observed across all microstates between the two groups. C, Occ. The Occ of microstate A was significantly higher in the LT group compared to the HT group. No significant differences in Occ were found for the other microstates. D, Cov. The Cov of microstate A was significantly greater in the LT group than in the HT group. For the remaining microstates, no

*significant differences in Cov were observed between the two groups. E, Based on a mean (5.55 ±1.76°) split of individual thresholds, participants were categorized into a high-threshold (HT, low-acuity) group (M = 7.00°, SD = 1.09°) and a low-threshold (LT, high-acuity) group (M = 4.07°, SD = 0.83°). *P < 0.05; **P < 0.01; ***P < 0.001.*

Transition probabilities: TP. To estimate the difference in the TP among the five microstate classes (A to E) between the HT and LT groups, we performed a 2 (group: HT vs. LT) × 20 (pair: A→B, A→C, A→D, A→E, B→A, B→C, B→D, E→A, etc.) mixed factor repeated-measures ANOVA with the observed probabilities as the dependent measure and sex as a covariate. Mauchly's test of sphericity was performed, and Greenhouse–Geisser corrected F-values and degrees of freedom are reported when sphericity was violated. The results showed a significant main effect of pair ($F_{19, 1482.00}$ = 47.27, p < 0.001, η^2_p = 0.38). Crucially, the interaction between group and pair was also significant ($F_{19, 1482.00}$ = 3.16, p < 0.001, η^2_p = 0.039). Post hoc tests (Tukey's HSD) indicated that the HT and LT groups differed significantly on the following pairs (Figure S3): pair C→E (t_{78} = 2.14, p = 0.036, *FDR-adjusted q = 0.13, Cohen's d* = 0.47), pair D→A (t_{78} = 2.50, p = 0.015, *FDR-adjusted q = 0.10, Cohen's d* = 0.30), pair D→B (t_{78} = 2.05, p = 0.044, *FDR-adjusted q = 0.13, Cohen's d* = 0.17), pair E→C (t_{78} = 3.12, p = 0.003, *FDR-adjusted q = 0.06, Cohen's d* = 0.77), pair A→D (t_{78} = 2.35, p = 0.021, *FDR-adjusted q = 0.11, Cohen's d* = 0.30), pair A→B (t_{78} = 2.53, p = 0.013, *FDR-adjusted q = 0.10, Cohen's d* = 0.37), pair B→D (t_{78} = 2.02, p = 0.047, *FDR-adjusted q = 0.13, Cohen's d* = 0.15).

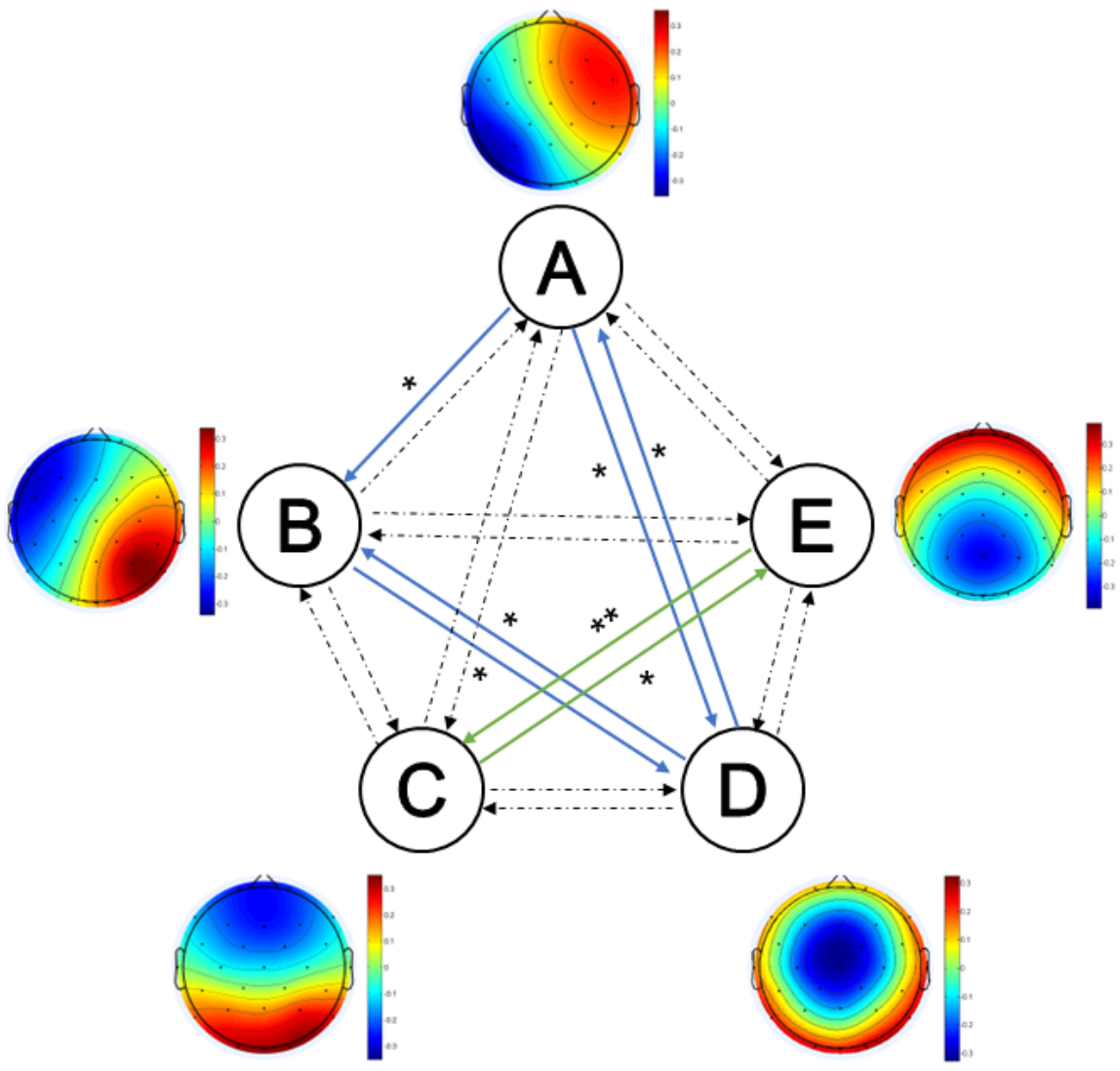

*Supplementary Figure S3. Results of TP among microstates. The black dashed line represents microstate pairs for which no significant difference in TP was found between the LT and HT groups. The blue solid line indicates pairs where TP was significantly greater in the LT group compared to the HT group, and the green solid line presents pairs where TP was significantly higher in the HT group than in the LT group. TPs were significantly elevated in the LT group relative to the HT group for microstate pairs A⟶B, A↔D, and B↔D. Conversely, TPs for pairs C↔E were significantly higher in the HT group compared to the LT group. *P < 0.05; **P < 0.01.*
